# Astragalin improves cognitive disorder in Alzheimer's disease: Based on network pharmacology and molecular docking simulation

**DOI:** 10.1111/cns.14799

**Published:** 2024-08-06

**Authors:** Rui Du, Hongyan Pei, Zhongmei He, Jin Wang, Xiaohong Zhou, Wenyan Li, Diwei Zhu, Caiqun Zhang

**Affiliations:** ^1^ College of Chinese Medicinal Materials Jilin Agricultural University Changchun China; ^2^ The Second Affiliated Hospital of Jiaxing University Jiaxing China

**Keywords:** Alzheimer's disease, astragalin, COX2, network pharmacology, neuroinflammation

## Abstract

We investigate the mechanism of action of astragalin (AST) in the treatment of Alzheimer's disease (AD). Network pharmacology was conducted to analyze the relationships among AST, AD, and neuroinflammation, The APP/PS1 transgenic mice with AD were used in the experiments; to be specific, the influence of AST on the behavior of mice was analyzed by Morris water maze and eight‐arm radial maze tests, the tissue inflammatory factor levels were detected by ELISA, and pathological changes were analyzed by H&E and immunohistochemical staining. Analysis results of network pharmacology suggested that AST exerted the multi‐target effect on neuroinflammation in AD. Through molecular docking and dynamics analyses, COX2 might be the target of AST. Moreover, animal experimental results demonstrated that AST improved the behavior of AD mice, and enhanced the motor and memory abilities, meanwhile, it suppressed the expression of inflammatory factors in tissues and the activation of microglial cells. this study discovers that AST can suppress microglial cell activation via COX2 to improve neuroinflammation in AD.

## Graphical abstract

By utilizing multiple methods, this study discovers that AST can suppress microglial cell activation via COX2 to improve neuroinflammation in AD, thereby improving the behavior of AD mice. Therefore, COX2 is an important target of AST.

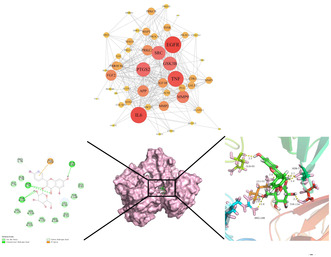

## 1 BACKGROUND

Astragalin (AST) is not only a derivative of kaempferol but also a natural flavonoid small molecule, which possesses diverse pharmacological effects such as anti‐inflammation, antitumor, anti‐oxidation, anti‐fibrosis, anti‐allergy, neuroprotection, and cardioprotection.^1^, ^2^ As discovered in current studies, AST involves multiple signals and targets small molecules, including transcription factor NF‐kB, protein kinase COX2, MMPs, and kinases JNK and P38.^3^, ^4^ Meanwhile, it can suppress inflammatory factors IL‐1β, IL‐6, and TNF‐α.^5^ It has been found in research on the nervous system that AST plays a vital role in degenerative disease and traumatic nerve injury; besides, it can improve the anti‐oxidative and anti‐inflammatory activities in rats with ischemic brain injury to exert its effect and suppress the apoptosis of neurons. In the meantime, it is discovered in the rat model of brain injury that, AST can suppress the expression of apoptins Bax and caspase‐3.^6^ Moreover, according to research at the cellular level, AST can inhibit the thapsigargin‐induced expression of endoplasmic reticulum stress‐related proteins to alleviate ischemic brain injury.^7^, ^8^ While AST possesses diverse pharmacological effects, its exact action target and mechanism remain to be further explored.

Alzheimer's disease (AD) is a kind of progressive degenerative disease involving multiple complex pathological processes.^9^ Recent research has found that neuroinflammation plays an important role in AD. AST exerts the neuroprotective effect on multiple traumatic nerve injuries,^10^, ^11^ but its role in AD is rarely reported, and its mechanism remains unclear. Therefore, using neuroinflammation as a starting point, this study was aimed at exploring the role and mechanism of AST in AD through combining several simulation means such as network pharmacology, molecular docking, and dynamics simulation with experiments.

## 2 MATERIALS AND METHODS

### 2.1 Network pharmacology analysis

The drug targets were predicted based on the PharmMapper and SwissTargetPrediction databases; meanwhile, the disease targets were acquired by the GeneCards, OMIM, and DISGENET databases. Then, a Venn diagram was plotted by the jvenn tool to obtain the intersection of disease targets and drug targets. Later, the effective active component‐target network of the drug was drawn based on the Cytoscape 3.8.2 database, and the intersected disease–drug target interaction network was constructed using the STRING platform. By utilizing the DAVID database, the intersected disease–drug targets were exposed to Gene Ontology (GO) and Kyoto Encyclopedia of Genes and Genomes (KEGG) pathway enrichment analyses. (1) To acquire the potential drug targets, targets of “Norm Fit ≥0.6” were selected from the PharmMapper prediction results, whereas targets of “Probability≥0.1” were chosen from SwissTargetPrediction, and the obtained targets were combined as the potential targets of AST after removing the duplicates. (2) Acquisition of potential disease targets: The databases “GeneCards,” “DisGeNET” and “OMIM” were searched with the keyword “Alzheimer's Disease” to obtain the potential disease targets. Typically, targets of “Relevance score≥upper quartile” were screened from the “GeneCards” database retrieval results, while those of “Score_gda≥0.1” were obtained from the “DisGeNET” database retrieval results; then, the results were combined with those obtained from other databases and the duplicates were removed to obtain the potential AD targets. (3) Construction of the “drug‐component‐disease target” network diagram: The Venn diagram was plotted with the jvenn tool to obtain the intersection between drug targets and disease targets as the potential drug targets for treating AD. The components and drugs corresponding to the intersected targets were screened, and the “drug‐component‐target” diagram was constructed with Cytoscape 3.8.2. Later, targets with Betweenness Centrality, Closeness Centrality, and Degree greater than the median values were selected as the hub nodes of the network. (4) Construction of the protein–protein interaction (PPI) network: By using the STRING platform, the disease–drug intersection target PPI network was constructed, the minimum interaction threshold was set at “highest confidence” > 0.4, the isolated nodes were concealed, and the rest parameters were defaults. Subsequently, the data were imported into Cytoscape 3.8.2 for network analysis. Nodes represented different targets, while edges stood for the relationships between different targets. (5) GO and KEGG pathway enrichment analyses: the disease–drug intersection targets were imported into the DAVID database (the functional annotation online tool) for GO enrichment analysis from three modules, namely, biological process, cellular component, and molecular function. Besides, KEGG pathway enrichment analysis was carried out. (6) Plotting of the KEGG pathway diagram: the disease–drug intersection targets were uploaded into the KEGG database, and the KEGG pathway diagram was plotted by the mapper tool.

### 2.2 Molecular docking and dynamics analyses

The 2D structures of small molecule ligands were obtained from the PubChem database (http://pubchem.ncbi.nlm.nih.gov/) and input into the Chem Office software to prepare their 3D structures, which were saved in the mol2 file. Subsequently, crystal structures with protein targets and relatively high resolution were selected based on the RCSB PDB database (http://www.rcsb.org/) as the molecular docking receptors. After dewatering and dephosphorylation of proteins with the PyMOL software, the proteins were saved in the PDB file. Moreover, energy minimization of the compounds was completed using the Molecular Operating Environment 2019 software, the target proteins were preprocessed, and the active pockets were searched. Finally, molecular docking was performed with MOE 2019. The binding activity of the two was evaluated according to the binding energy, and the results were visualized with PyMOL and Discovery studio software.

### 2.3 Mouse model

The wild‐type C57BL/6J mice and 10‐month‐old APP/PS1 double transgenic AD mice (AD) were purchased from the Nanjing Institute of Model Animals and bred in our laboratory. The C57BL/6J mice were defined as the normal control group (Control), while AD mice were divided into AD and AST groups, with the AST group being further classified as AST‐L (50 mg/kg), and AST‐H (100 mg/kg) groups. Mice were given intragastric administration of AST or equivalent amount of normal saline once a day for 5 weeks consecutively, and there were six mice in each group. Neurobehavioral tests were conducted in mice after 5 weeks, and mice were sacrificed by carbon dioxide suffocation.

Our animal experimental protocols were reviewed and approved by the Ethics Committee of Jiaxing University. Animal experiments complied with the animal ethics and welfare regulations. All mice were raised in the same environment.

### 2.4 Morris water maze test

The platform was set at around 1 cm above the water level.^12^ In the learning stage, each mouse has conducted four tests for learning every day. The platform was placed at two different positions in the first two and the latter two experiments, and the mouse was placed at the lateral position of the quadrat opposite to the platform. The mouse was allowed for free exploration in water for 1 min; if the mouse could not find the platform within 1 min, it was guided to the platform and stayed there for 15 s. Later, the mouse was dried, and the time interval between the two experiments of one mouse should be no less than 20 min. A certain time after the completion of the platform conceals experiment (the precise time interval was determined according to the experimental objective), the platform was removed, and the mouse was placed from position 1 (the position farthest to the target platform which was not used in the platform concealing stage). The mouse travel process was recorded for subsequent analysis.

### 2.5 Eight‐arm radial maze test

In the training stage, the mouse was placed in the center of the eight‐arm maze, and there was a food reward point in the terminal of each arm.^13^ During the first several tests, the mouse was allowed for free exploration in the maze to get familiar with the environment. Subsequently, the reward points were gradually introduced, and the frequencies of the mouse entering each arm and obtaining reward were recorded. In the test stage, food rewards were removed from some arms in the maze, and the concept of working memory was introduced. The frequency of working memory error of the mouse in each test was recorded. In the intervention stage, one arm of the maze was closed and specified as the novel arm to test the reference memory. After a certain period of time, the mouse was introduced into the maze again to observe whether it could remember the unexplored novel arm. The frequency of the mouse entering the novel arm was recorded and compared with other arms to evaluate its spatial reference memory. The frequency of working memory error was defined as the frequency of the mouse re‐entering the previously visited arm. The frequency of reference memory error was defined as the frequency of the mouse entering the non‐reward arm, especially the novel arm. Travel distance was the total travel distance of the mouse in the maze. Detection time was the time taken by the mouse to complete the maze task.

### 2.6 Enzyme‐linked immunosorbent assay (ELISA)

The expression levels of inflammatory factors IL‐6, IL‐1β, and TNF‐α in mouse brain tissue were detected. To be specific, the mouse brain tissue was grinded in liquid nitrogen till there was no granule, and then, 1 mL NP‐40 lysate (Beyotime Biotechnology Co., Ltd, Shanghai, China) was added for lysis on ice for 30 min, and the supernatant protein liquid was collected for use. In cell experiments, the culture medium was collected and preserved at −80°C. After obtaining all samples, the samples were centrifuged at 3000 rpm for 20 min, and the supernatant was collected for detection following the ELISA kit instructions (Jiancheng Bioengineering Institute, Nanjing, China). The results were calculated using the standard curve method.

### 2.7 H&E and immunohistochemical staining

After sacrifice, mice were subjected to cerebral perfusion. The mouse brain tissue was prepared into the 4‐μm serial freezing sections for H&E staining using the H&E staining kit (Beyotime Biotechnology Co., Ltd, Shanghai, China) instructions. The sections were stained with hematoxylin for 3 min, rinsed with tap water for 2 min, and treated with 1% hydrochloric alcohol for 2 s. After washing with tap water for 2 min again, the sections were treated with 1% ammonium hydroxide for 20 s, then with 0.5% eosin alcohol for 10 s, dehydrated with gradient alcohol, transparentized with xylene, and mounted with neutral balsam. Finally, the brain pathological changes were observed under the light microscope.

The mouse brain tissue was fixed with 4% paraformaldehyde, dehydrated with sucrose solution, embedded in OCT, and prepared into sections. The sections were later washed with PBS, blocked with 5% serum for 30 min, and incubated with IBA‐1 monoclonal antibody (Abcam, Massachusetts, USA) diluted with TBST solution at 1:100 overnight at 4°C. After washing with PBS three times, the Peroxidase Substrate Kit (Abcam, Massachusetts, USA) was adopted for color developing, and sections were observed under the microscope.

### 2.8 Statistical analysis

All measurement data were expressed as mean ± standard deviation ($\bar{x}$ ± *s*), and data between the two groups were compared by two independent sample *t*‐test, while data among three or more groups were explored by one‐way ANOVA. The subsequent pairwise comparisons between the two groups were conducted with LSD. All the above‐mentioned tests were two‐sided, and *p* < 0.05 stood for statistical difference.

## 3 RESULTS

### 3.1 Network pharmacology analysis results

**FIGURE 1:**
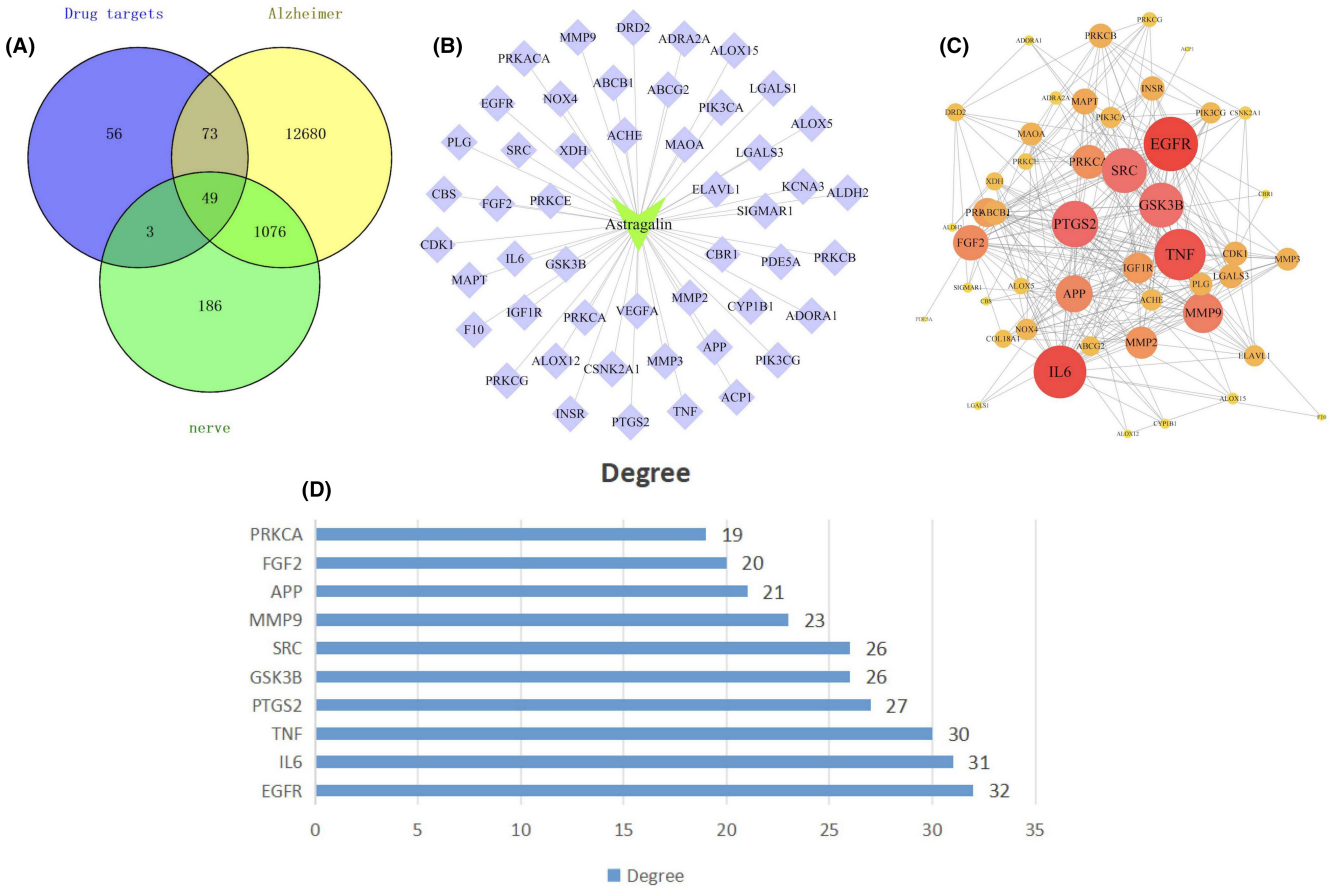
Analysis of astragalin (AST)‐Alzheimer's disease (AD)‐neuroinflammation targets. (A) Target intersection analysis results; (B) AST target analysis results; (C) protein–protein interaction (PPI) network analysis results of the target relationships; (D) AST dominant target analysis results, with EGFR, SRC, COX2, and GSK‐3β being the dominant targets.

**FIGURE 2:**
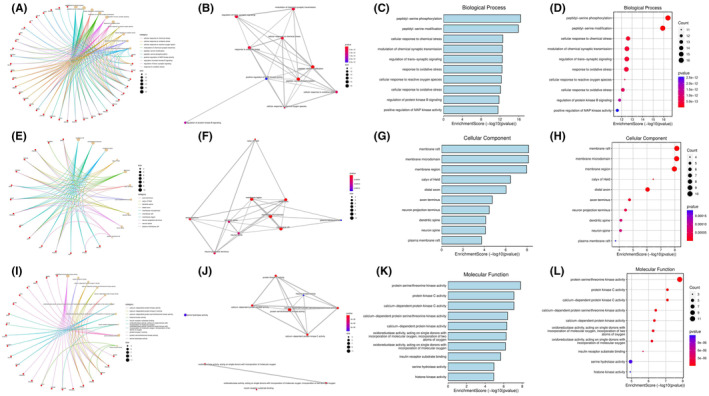
Gene Ontology (GO) analysis results of astragalin (AST)‐Alzheimer's disease (AD)‐neuroinflammation. (A–D) Biological process analysis results. (E–H) Cellular component analysis results. (I–L) Molecular function analysis results. 10 most closely associated signals were displayed with topology analysis.

**FIGURE 3:**
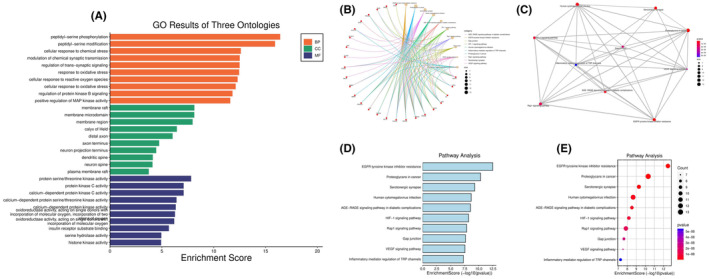
Kyoto Encyclopedia of Genes and Genomes (KEGG) analysis results. (A) Combined display of Gene Ontology (GO) analysis results. (B–E) KEGG analysis results.

The targets of AST, AD, and neuroinflammation were searched and intersected to obtain a total of 49 common targets among the three [Figure 1A]. The core targets were visualized and associated with AST [Figure 1B]. The relationships between targets were displayed as the PPI network, among which, the core targets were EGFR, SRC, COX2, GSK‐3β, and inflammatory factors [Figure 1C]. The targets were analyzed for their Degree levels, and the top 10 targets with the closest association were displayed [Figure 1D]. In GO analysis, we analyzed the AST‐related BPs, CCs, and MFs. As a result, AST was related to multiple BPs, among which the 10 most closely associated signals were displayed with topology analysis and bubble diagram [Figure 2A–D]. Similarly, CCs [Figure 2E–H] and MFs [Figure 2I–L] were analyzed and displayed. After the combined display of GO analysis, the enrichment scores were formed [Figure 3A]. The AST‐related signaling pathways were analyzed by KEGG, and the top 10 most closely related signaling pathways were displayed by topology analysis and bubble diagram [Figure 3B–E].

### 3.2 Improvement of AST in the cognitive behavior of AD mice

**FIGURE 4:**
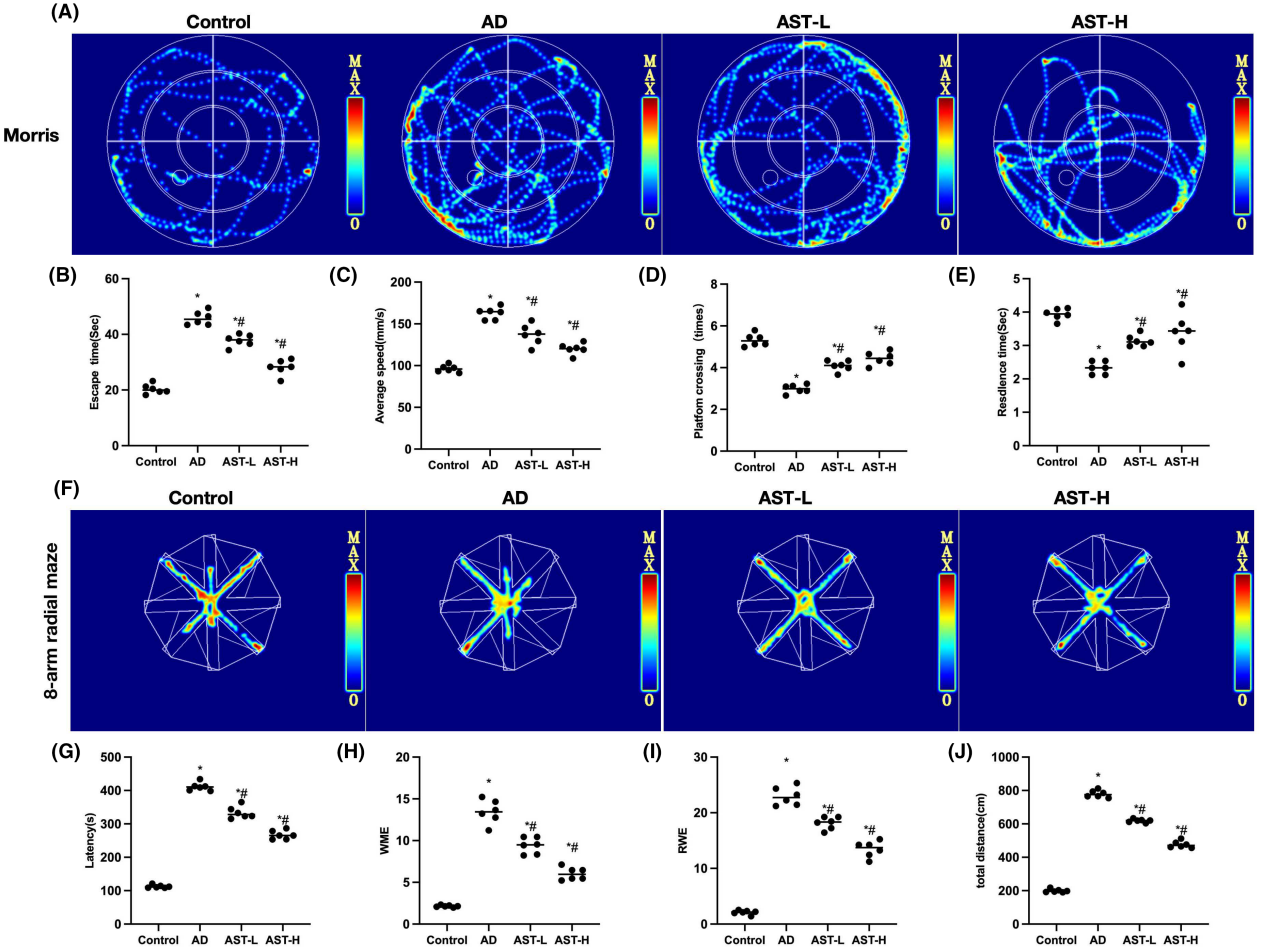
Improvement of astragalin (AST) on cognitive disorder in Alzheimer's disease (AD) mice. (A–E) Morris water maze test (*n* = 6). The escape latency and average speed of mice in the AD group were significantly higher than those in the Control group, while the platform crossing frequency and time were apparently lower than those in the Control group. AST could enhance the cognitive disorder in mice, and reduce the escape latency, average speed, platform crossing frequency, and time, in a dose‐dependent manner. (F–J) Eight‐arm radial maze test (*n* = 6). The escape latency and total travel distance of mice in the AD group were higher than those in the Control group, and AST reduced the escape latency and total travel distance. *p < 0.05, compared with the Control group; ^#^ *p* < 0.05 compared with the AD group.

As suggested by Morris water maze test results, the escape latency and average speed of mice in the AD group were significantly higher than those in the Control group, while the platform crossing frequency and time were significantly lower than those in the Control group. AST could improve the cognitive disorder in mice, and reduce the escape latency, average speed, platform crossing frequency, and time, in a dose‐dependent manner [Figure 4A–E]. Eight‐arm radial maze test results also demonstrated that the escape latency and total travel distance of mice in the AD group were higher than those in the Control group, and that AST lowered the escape latency and total travel distance [Figure 4F–J].

**FIGURE 5:**
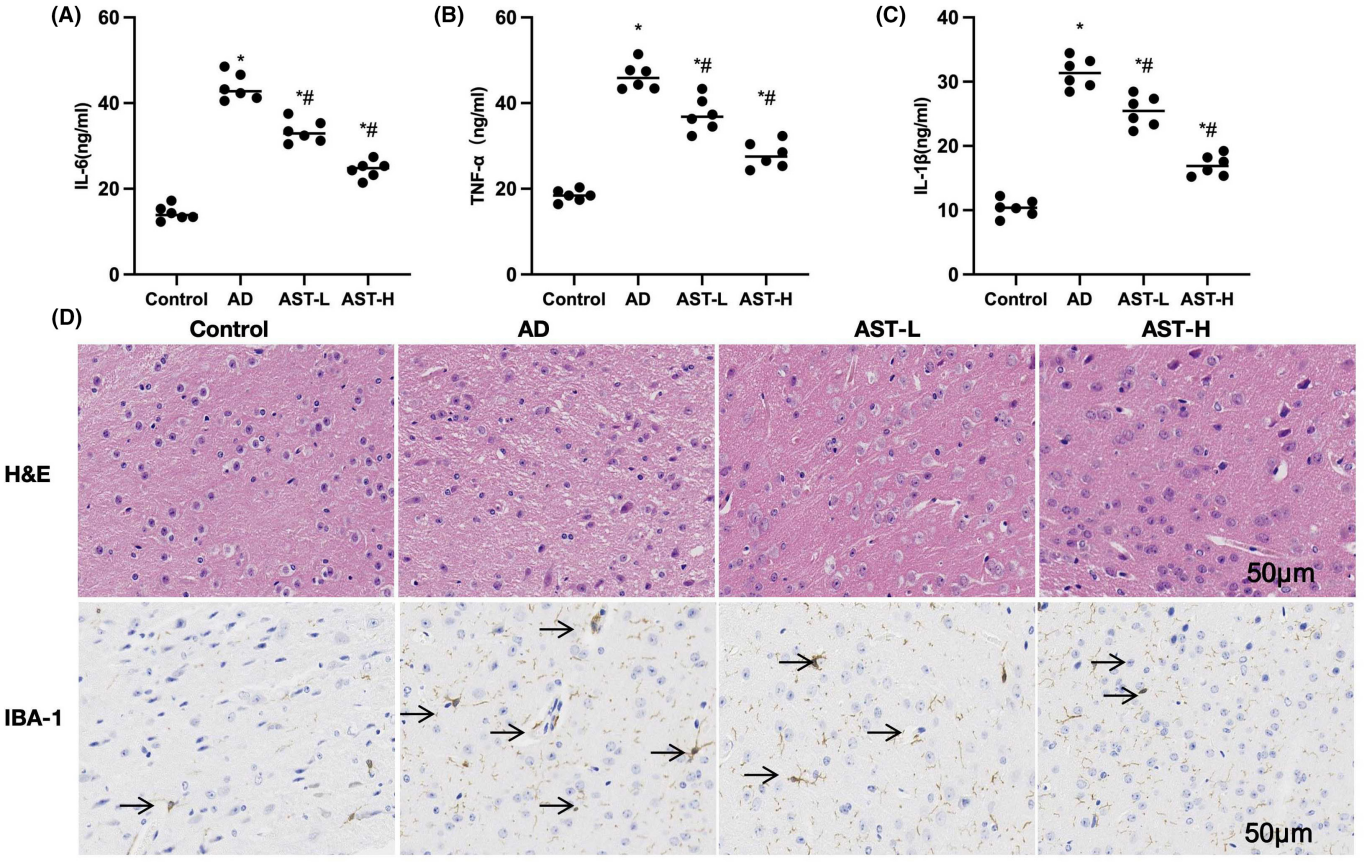
Effect of astragalin (AST) on pathological changes in Alzheimer's disease (AD) mice. (A–C) ELISA (*n* = 6). The levels of inflammatory factors IL‐6, IL‐1β, and TNF‐α in the brain tissue of AD mice were apparently higher than those in the Control group, and AST decreased the inflammatory factor levels in brain tissue dose‐dependently. (D) H&E and IHC staining (*n* = 6). H&E staining results demonstrated the presence of cell apoptosis and certain inflammatory responses in the AD group, while AST reduced tissue inflammatory responses, and suppressed cell apoptosis. Based on IHC staining results, the IBA‐1‐positive cell number in the AD group was markedly higher than that in the Control group, and the IBA‐1 expression in the AST group was significantly down‐regulated. *p < 0.05, compared with the Control group; ^#^ *p* < 0.05 compared with the AD group.

According to ELISA results, the levels of inflammatory factors IL‐6, IL‐1β, and TNF‐α in the brain tissue of AD mice were apparently higher than those in the Control group, and AST decreased the inflammatory factor levels in brain tissue dose‐dependently [Figure 5A–C]. H&E staining results demonstrated the presence of cell apoptosis and certain inflammatory responses in the AD group, while AST reduced tissue inflammatory responses, and suppressed cell apoptosis. Based on IHC staining results, the IBA‐1‐positive cell number in the AD group was markedly higher than that in the Control group, and the IBA‐1 expression in the AST group was significantly down‐regulated [Figure 5D].

### 3.3 Molecular docking results of AST and core target proteins

**FIGURE 6:**
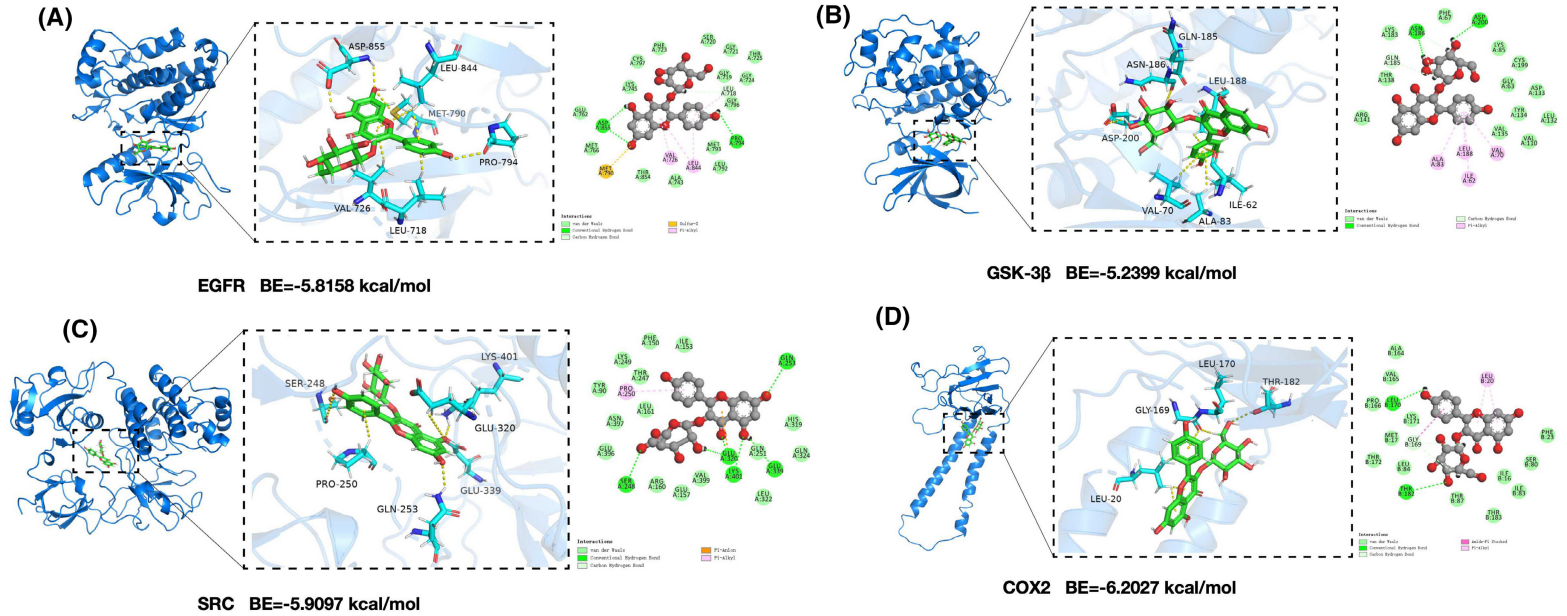
Molecular docking results. (A–D) Docking simulation results between astragalin (AST) and core target proteins EGFR, GSK‐3β, SRC, and COX2.

The Asp855, Pro794, and Leu718 residues on the EGFR receptor formed the hydrogen bond interactions with AST. The Val726, Leu844, and Leu718 residues formed the hydrophobic interactions with AST. Additionally, the Met790 residue formed the Sulfur‐X interaction force with AST. The Leu170, Thr182, and Gly169 residues on the COX2 receptor formed the hydrogen bond interactions with AST. The Leu20 and Gly169 residues formed the hydrophobic interactions with AST. The Ser248, Glu320, Lys401, Glu339, and Gln253 residues formed the hydrogen bond interactions with AST. The Pro250 residue formed the hydrophobic interaction with AST. Moreover, the Glu320 residue formed the electrostatic interaction force with AST. The Gln185, Asn186, and Asp200 residues on the GSK‐3β receptor formed the hydrogen bond interactions with AST. The Ala83, Leu188, Ile62, and Val70 residues formed the hydrophobic interactions with AST [Figure 6A–D].

### 3.4 Molecular dynamics simulation results of AST and COX2

**FIGURE 7:**
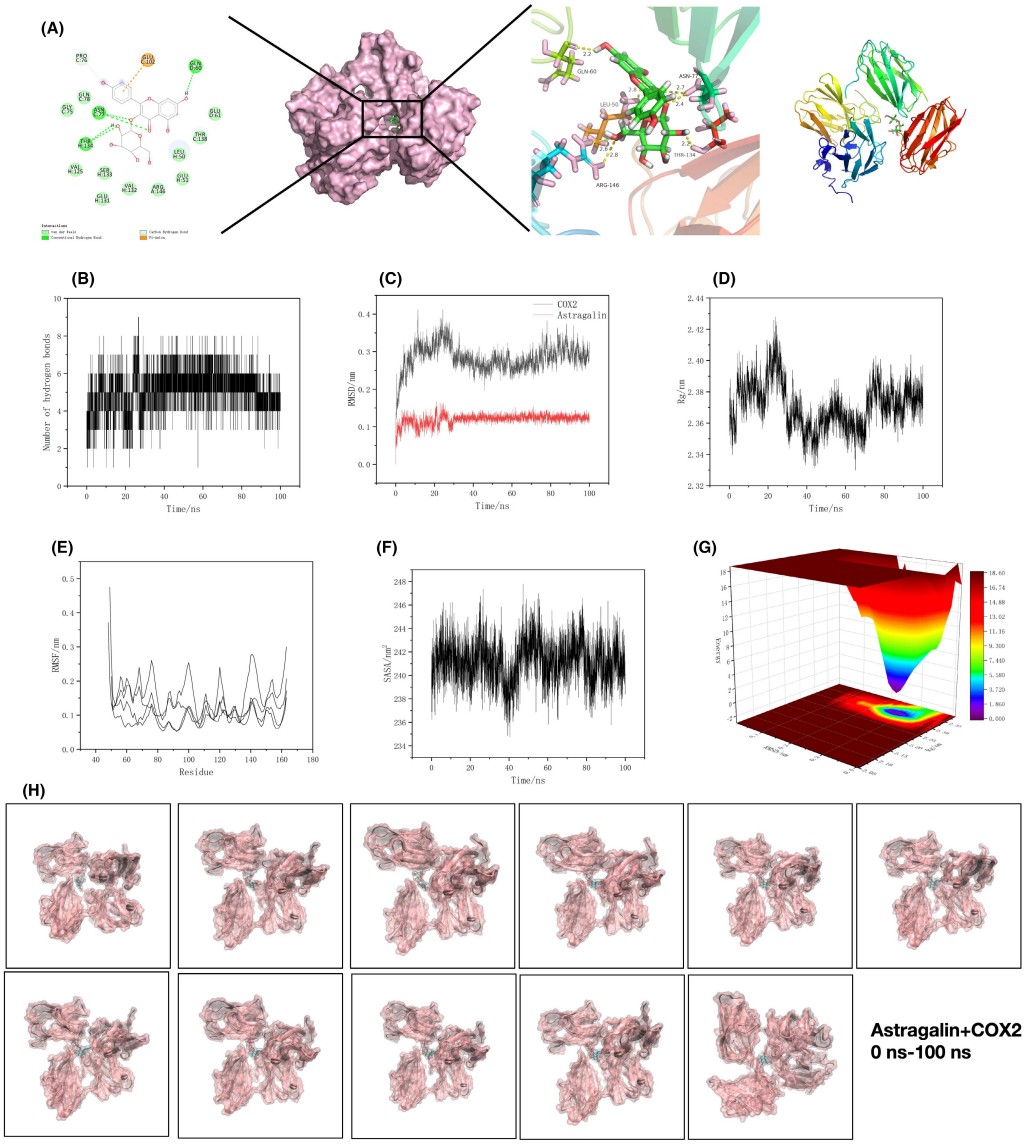
Dynamics analysis between astragalin (AST) and COX2. (A) Molecular docking pattern. (B) Hydrogen bonding pattern analysis results. (C) RMSD analysis results. (D) Rg analysis pattern. (E) SASA analysis results. (F) RMSF analysis results. (G) Energy trap analysis results. (H) Conformational changes in 0–100 ns.

The binding energy between COX2 and AST was the lowest; in the meantime, COX2 was an important regulatory protein of microglial cell activation. Therefore, we conducted dynamics analysis after binding AST to COX2. After molecular docking between COX2 and AST, the 2D and 3D results were visualized [Figure 7A]. The binding was subjected to dynamics analyses including hydrogen bonding [Figure 7B], RMSD [Figure 7C], and Rg [Figure 7D], and the results revealed the stable bonding pattern between the two. Moreover, as revealed by SASA [Figure 7E], RMSF [Figure 7F], and energy trap [Figure 7G] analyses, there was a low‐energy binding pattern between COX2 and AST, which was the stable binding model. Finally, we analyzed the conformational changes in 0–100 ns [Figure 7H].

## 4 DISCUSSION

AST possesses extensive biological activities and therapeutic effects, and its pharmacological properties include anticancer, anti‐inflammation, anti‐oxidation, neuroprotection, cardioprotection, anti‐ulcer, and anti‐fibrosis.^13^ Diverse in vivo and in vitro studies on AST has illustrated its medicinal properties and mechanism of action.^14^ The role of AST in anti‐inflammation has been reported. Inflammation is the direct response of the body to tissue injuries induced by toxic stimuli like pathogens and physical or chemical injuries.^15^ While inflammatory response is a kind of defense mechanism, its persistent existence may result in multiple pathological symptoms, and AST can down‐regulate the NF‐κB signaling pathway to suppress inflammatory responses.^16^, ^17^ Research has confirmed that AST can suppress the lipopolysaccharide (LPS)‐induced inflammatory mediators. AST significantly decreases the LPS‐induced expression of iNOS, COX‐2, and cytokines/chemokines, dramatically decreasing the NO production in macrophages of J774A.1 mice, and suppresses the LPS‐induced NF‐κB activation.^18^ It is found from in vivo experiments that, in the mouse model, AST down‐regulates the NF‐κB signaling pathway in the LPS‐induced mastitis to suppress the inflammatory responses.^19^ Similarly, some in vivo studies also demonstrate that AST pretreatment can increase the survival rate of the fatal endotoxemia, and weaken the inflammatory responses in the mouse model of LPS‐induced acute lung injury.^20^ The anti‐inflammatory mechanism of AST is related to suppressing the tumor necrosis factor‐α (TNF‐α), interleukin‐1 (IL‐1), and interleukin‐6 (IL‐6) through inactivating NF‐κB.^21^

As discovered from research on AD, neuroinflammation is an important pathological factor promoting the occurrence and development of AD, among which the activation of microglial cells and the expression of inflammatory factors are the keys to neuroinflammation.^22^ COX2 is a vital mediating protein of neuroinflammation. Microglial cells are excessively activated in cognitive disorder.^23^ There have been multiple inflammatory factors detected in neurogliocytes; For instance, the contents of TNF‐α, IL‐1β, and IL‐6 increase, which will recruit a large amount of immune cells to attack the blood–brain barrier (BBB) and increase the BBB permeability.^24^ In the meanwhile, the up‐regulation of inflammatory factors will influence the absorption and release of neurotransmitters and the function of neurons. COX2 can also trigger the conversion of arachidonic acid into prostaglandins (PGs). PG E2 acts on four G protein‐coupled receptors in the brain, among which EP1 is the most closely related to BBB injury, and the EP2 receptor indirectly participates in the mechanism of neurotoxicity.^25^ It is found in research that both suppressing COX‐2 and blocking the PG E2 receptor can protect the neurons and suppress cognitive disorder. The COX2‐mediated microglial cell activation is also a vital mechanism of neuroinflammation, inflammatory factors can be secreted after the excessive activation of microglial cells, which can further amplify the inflammatory cascade and scope, thereby persistently affecting neuronal injury.^26^, ^27^

In this study, we adopted network pharmacology methods to analyze the direct relationships among AD, neuroinflammation, and AST. Through PPI network construction, EGFR, SRC, COX2, and GSK‐3β were identified as the possible targets of AST. At the same time, AST involved multiple signals. In our research on the AD mouse model, AST was found to improve cognitive disorder in AD mice and suppress inflammatory factor expression in tissues and microglial cell activation. IBA‐1 is the major marker protein of microglial cell activation, and the decreased number of IBA‐1‐positive cells suggested that AST hindered the activation of microglial cells. Molecular docking results also proved the lower binding energy between AST and COX2. Furthermore, dynamics simulations also revealed the stable binding conformation between AST and COX2, consistent with experimental results. At present, the pathological and physiological differences between female and male mice in AD research have been revealed, but this study did not further explain the impact of AST on AD mice of different genders. This is a limitation of this study and also a direction for future research.

## 5 CONCLUSION

Through network pharmacology, molecular docking, dynamics simulations, and experiments, results in this study demonstrate that AST can improve cognitive disorder and neuroinflammation in AD mice, and its mechanism of action may be associated with suppressing the COX2‐mediated microglial cell activation. AST is a potential small molecule for the treatment of AD.

## FUNDING INFORMATION

This study was supported by the Zhejiang Medical and Health Plan Project [2022KY1259] and basic public welfare projects in Zhejiang Province [LGD22H160013].

## CONFLICT OF INTEREST STATEMENT

No competing interests.

## CONSENT FOR PUBLICATION

All authors approved to publish the article.

## Data Availability

The data that support the findings of this study are available from the corresponding author upon reasonable request.
